# The complete mitochondrial genome of *Proedromys bedfordi* Thomas 1911 (Arvicolinae, Rodentia)

**DOI:** 10.1080/23802359.2024.2422978

**Published:** 2024-12-08

**Authors:** Shiqing Wang, Chen Lin, Zhen Wang, Zhangwen Deng

**Affiliations:** a College of Life Sciences, University of Chinese Academy of Sciences, Beijing, China; b College of Wildlife and Protected Area, Northeast Forestry University, Harbin, China; c Guangxi Zhuang Autonomous Region Forest Inventory and Planning Institute, Nanning, China

**Keywords:** *Proedromys*, mitogenome, phylogenetic analysis

## Abstract

The Duke of Bedford’s vole (*Proedromys bedfordi* Thomas 1911) is distributed only at the border areas of the Sichuan and Gansu Provinces, China. In this study, the first complete mitochondrial genome of *P. bedfordi* is generated and characterized. The assembled genome is 16,262 base pairs in length and the base compositions present clearly the A-T bias (60.84%). Its genetic constitution and arrangement are consistent with the taxon of the voles, including 13 protein-coding genes, 22 tRNA genes, 2 rRNA genes, and 2 main non-coding regions, D-loop region and O_L_ region. This mitochondrial genome will be a significant supplement for the genus *Proedromys* and whole mitogenome phylogenetic analysis provided insights into further evolutionary research of the subfamily Arvicolinae.

## Introduction

*Proedromys bedfordi* Thomas 1911 belongs to the Rodentia order, Cricetidae family, Arvicolinae subfamily, Microtini tribe, genus *Proedromys* (Chen et al. 2012; Upham et al. 2023). This species is found in the border regions of Sichuan and Gansu Provinces in China. Due to its narrow range of occurrence and threats such as habitat destruction by human activities, it is listed as a vulnerable species by IUCN (Johnston 2016). In the context of the rapid global decline in biodiversity, the conservation and protection of endangered species have become particularly urgent, while the most important and direct indicator for measuring the evolutionary potential of endangered species is their genetic diversity (Frankham et al. 2010). Therefore, obtaining genomic data of *Proedromys bedfordi* is crucial for understanding its genetic diversity and implementing precise conservation. However, due to the scarcity of samples, GenBank currently contains only a few mitochondrial *COI* and *Cytb* sequences, along with limited nuclear DNA data for *P. bedfordi.*

Here, we report the first complete mitogenome of *P. bedfordi*, providing data support and molecular evidence for the phylogenetic relationship of *P. bedfordi* with other species in Arvicolinae. This not only offers valuable genetic resources for the protection of *P. bedfordi*, but also promotes further development in biodiversity conservation, thereby supporting the sustainability of global natural resources.

## Materials

**Figure 1.**
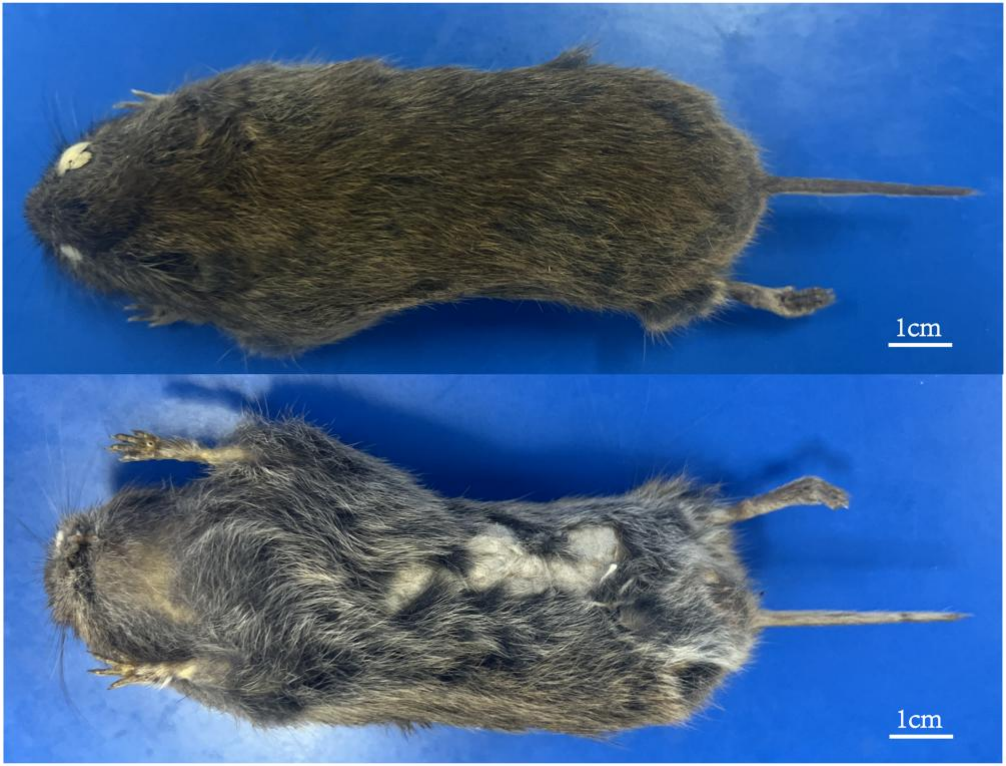
The image of *Proedromys bedfordi* (voucher no.: J49001). Photo by Chen Lin. The top image shows the dorsal view, while the bottom image shows the ventral view, with the belly filled with cotton.

A male specimen of *P. bedfordi* was collected from Liujiaping Township, Wen County, Gansu Province, China (32.94°N, 104.68°E) (Figure 1). Sterile tools were used for the collection of skin tissue, and the sample was immediately preserved and stored at −20 °C upon arrival at the laboratory to maintain DNA integrity. The specimen is stored at the Forensic Identification Institute of Northeast Forestry University (contact: Zhen Wang, 25252170@qq.com) under the voucher number J49001. The specimen is distinguished by its gray-brown dorsal fur, grayish white ventral fur, a medium-length tail that is gray-brown above and dull white below, and yellow incisors with distinct grooves (Liu et al. 2007). Based on its morphological characteristics and geographical distribution, the collection was identified by Zhen Wang.

## Methods

The total genomic DNA was extracted from the *P. bedfordi* skin tissue using TIANamp Micro DNA Kit (TIANGEN, Beijing, China; DP316). The DNA libraries were sequenced by the DNBSEQ-T1 sequencer (BGI, China), and raw sequencing reads were filtered using Trimmomatic (v0.39) (Bolger et al. 2014) with the parameters ‘*ILLUMINACLIP:adapter_BGI.fa:2:30:10 LEADING:35 TRAILING:35 MINLEN:60’* to obtain clean reads. NOVOPlasty (v.4.3.1) (Dierckxsens et al. 2017) was used for assembly and mitogenome annotation was performed in Geseq (Tillich et al. 2017). Clean reads were then mapped to the assembled mitogenome using Burrows-Wheeler Aligner (BWA) with the *mem* algorithm (v0.7.17-r1188) (Li and Durbin 2010). The bam file was filtered using SAMtools (v1.15) (Danecek et al. 2021) to calculate the coverage depth, with parameters ‘*samtools view -b -F 4 -F 256 -F 1024*’ to filter out unmapped, secondary, and duplicate reads. Reads with *NM:i* values > 2 were removed using awk command, and ‘*samtools depth*’ was used to calculate coverage depth. The circular mitogenome map was drawn using Chloroplot (Zheng et al. 2020) and the base composition was analyzed by MEGA 11 (Koichiro et al. 2021). Twenty-four mitogenomes of species belonging to the subfamily Arvicolinae were selected by conducting GenBank BLAST searches using the assembled *P. bedfordi* mitogenome and incorporating data used in phylogenetic analyses from published studies. The 13 protein-coding genes (PCGs) were extracted and aligned using MEGA 11 (Koichiro et al. 2021) and IQ-TREE (v.2.3.0) (Lam-Tung et al. 2015) was used to reconstruct the maximum-likelihood (ML) phylogeny based on 13 PCGs from 24 species of Arvicolinae, with the lesser long-tailed hamster (*Cricetulus longicaudatus*) as an outgroup. Phylogenetic analysis was run with 1,000 bootstrap replications, and the tree was visualized by iTOL (Letunic and Bork 2024). The best nucleotide substitution model (GTR+F + R5) was selected by comparing Bayesian Information Criterion (BIC) scores (Posada and Buckley 2004) using jModelTest (v.2.1.10) (Darriba et al. 2012).

## Results

**Figure 2.**
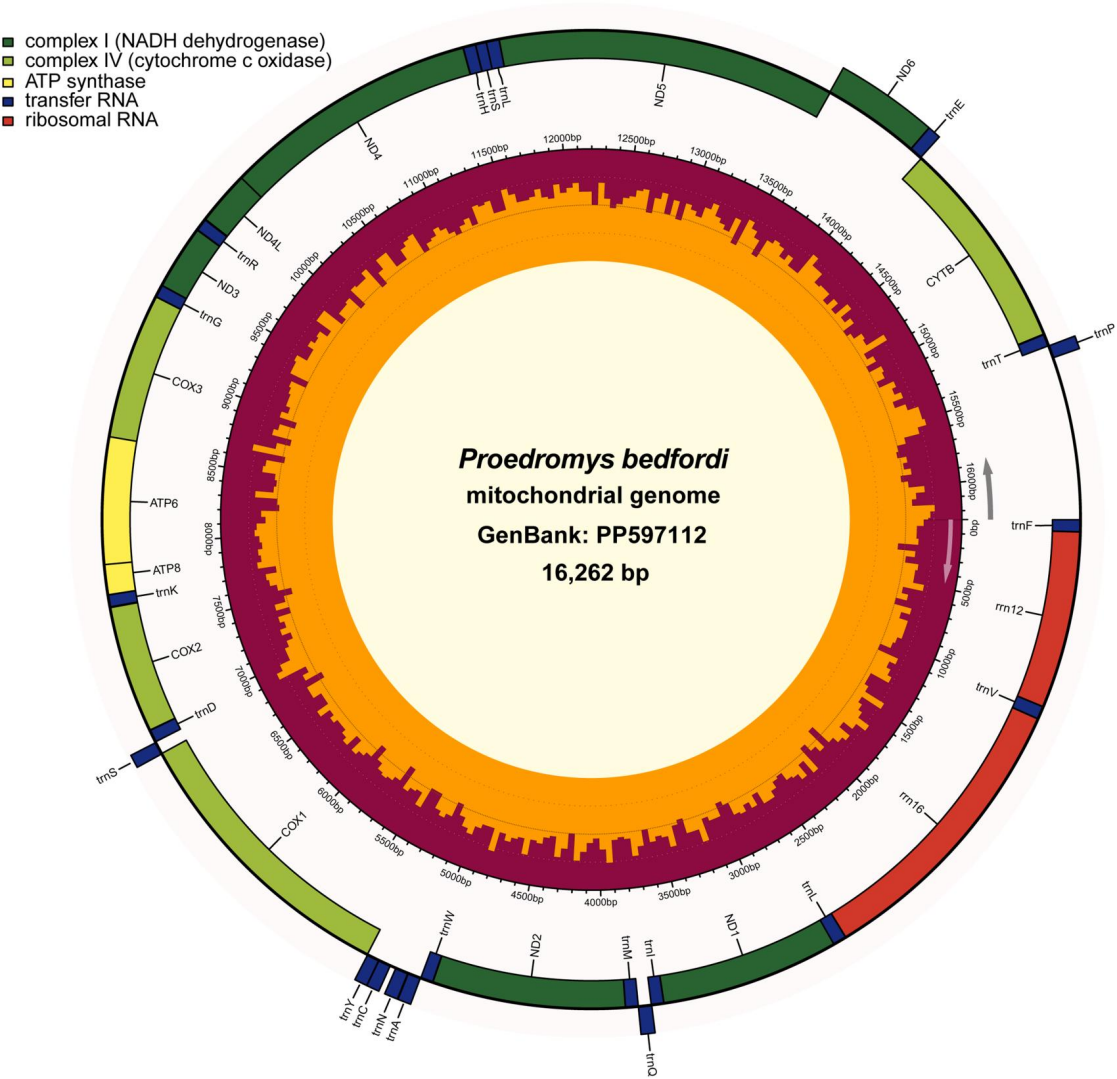
Mitochondrial genome map of *Proedromys bedfordi*. The innermost rings of the image depict the GC content and longer lines indicate higher %GC.

**Figure 3.**
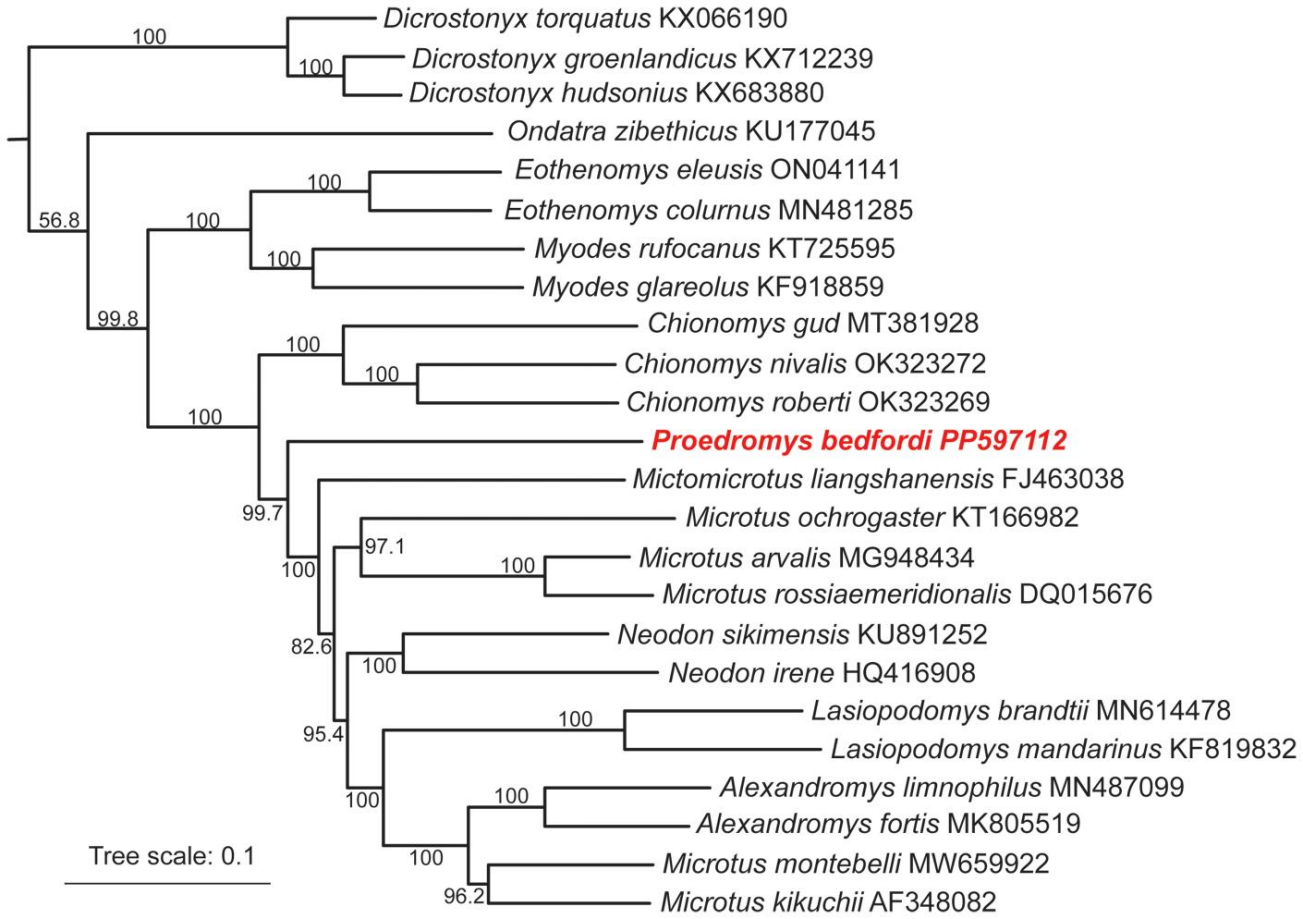
Maximum-likelihood tree of the subfamily Arvicolinae based on the sequences of 13 protein-coding genes (the outgroup is not shown for graphical reason). Numbers beside each node correspond to ML bootstrap values and the scale bar represents nucleotide substitutions per site. The following sequences were used: *Cricetulus longicaudatus* (KM067270) (Zhang et al. 2016b), *Dicrostonyx groenlandicus* (KX712239) (Fedorov and Goropashnaya 2016b), *Dicrostonyx hudsonius* (KX683880) (Fedorov and Goropashnaya 2016b), *Dicrostonyx torquatus* (KX066190) (Fedorov and Goropashnaya 2016a), *Myodes rufocanus* (KT725595) (Lu et al. 2017), *Myodes glareolus* (KF918859) (Bendová et al. 2016), *ondatra zibethicus* (KU177045) (Zhao et al. 2018), *Eothenomys colurnus* (MN481285) (Liu et al. 2022), *Eothenomys eleusis* (ON041141) (Zhu et al. 2023), *Alexandromys limnophilus* (MN487099) (Liu et al. 2022), *Alexandromys fortis* (MK805519) (Zhu et al. 2019), *Microtus montebelli* (MW659922) (Sogabe et al. 2021a), *Microtus kikuchii* (AF348082) (Lin et al. 2002), *Microtus rossiaemeridionalis* (DQ015676) (Triant and DeWoody 2006), *Microtus ochrogaster* (KT166982) (Cao et al. 2016), *Microtus arvalis* (MG948434) (Folkertsma et al. 2018), *Neodon irene* (HQ416908) (Fan et al. 2011), *Neodon sikimensis* (KU891252) (Zhang et al. 2016a), *Lasiopodomys mandarinus* (KF819832) (Cong et al. 2016), *Lasiopodomys brandtii* (MN614478) (Tian et al. 2020), *Chionomys nivalis* (OK323272) (Şeker et al. 2022), *Chionomys roberti* (OK323269) (Şeker et al. 2022), *Chionomys gud* (MT381928) (Abramson et al. 2021), *Mictomicrotus liangshanensis* (FJ463038) (Hao et al. 2011) and *Proedromys bedfordi* (PP597112).

The total length of the mitogenome of *P. bedfordi* is 16,262 bp and contained one control region (CR), two ribosomal RNA (rRNA), 22 transfer RNA (tRNA), 13 protein-coding genes (PCGs), and the origin of L-strand replication (O_L_R) (Figure 2). Depth of coverage map of mitogenome is shown in Supplementary Figure S1. Nucleotide composition is as follows: A, 32.81%; C, 25.93%; G, 13.23%; T, 28.03%. Nine PCGs (*ATP6*, *ATP8*, *COX1*, *COX2*, *COX3*, *Cytb*, *ND4*, *ND4L, ND6*) start with the ATG, three (*ND1*, *ND3*, *ND5*) used ATA, and *ND2* start with ATT. As for the stop codons, ten PCGs have either TAA or TAG as a stop codon, and the other three genes (*COX3, ND1, ND4*) end with a T residue. Our phylogenetic analysis (Figure 3) shows that species of the same genus cluster together. High bootstrap values (99.7%) separate *P. bedfordi* from *Mictomicrotus liangshanensis*, and the mitogenome of *P. bedfordi* is closely related to those of the genera *Chionomys*, *Mictomicrotus*, and *Microtus*.

## Discussion and conclusions

In this study, we first report the mitogenome of *P. bedfordi* and give a detailed annotation for this genome. The research findings indicate that the mitogenome of *P. bedfordi* shares similarities in terms of nucleotide composition, and genome structure with other reported species within the subfamily Arvicolinae (Bondareva et al. 2020; Sogabe et al. 2021b). Additionally, previous research identified *Proedromys liangshanensis* as a new species in the genus *Proedromys* (Liu et al. 2007), but morphological and phylogenetic studies reclassified *liangshanensis* into a recently designated monotypic genus named *Mictomicrotus* (Withnell and Scarpetta 2024). Our phylogenetic results also support that *P. bedfordi* is not a sister taxon to *Mictomicrotus liangshanensis*, with high bootstrap values separating the two despite their close relationship. Therefore, this study represents the first mitogenome in the genus *Proedromys*. The mitogenome data of *P. bedfordi* is not only crucial for studies of ecology conservation for this species, but also significantly enhances our understanding of the phylogenetic relationships and evolutionary history of this genus, as well as its related groups.

## Supplementary Material

Supplemental Material

## Data Availability

The genome sequence data is available in GenBank at https://www.ncbi.nlm.nih.gov under the accession number PP597112. The associated BioProject, SRA and Bio-Sample numbers are PRJNA1107982, SRR28961178, and SAMN41219189, respectively.
