# Supplementary material for: The complete mitochondrial genome of Proedromys bedfordi Thomas 1911 (Arvicolinae, Rodentia)
Source: Mitochondrial DNA B Resour. 2024 Dec 8;9(12):1653–7. doi: 10.1080/23802359.2024.2422978 (PMC11632922; doi:10.1080/23802359.2024.2422978)
Supplement: Supplemental Material [file TMDN_A_2422978_SM0858.pdf]

Supplementary materials

The complete mitochondrial genome of *Proedromys bedfordi* Thomas 1911 (Arvicolinae, Rodentia)

Shiqing Wang[a], Chen Lin[b], Zhen Wang[b], Zhangwen Deng[c]

[a]College of Life Sciences, University of Chinese Academy of Sciences, Beijing 100049, China; SW (shiqingwang08@gmail.com, ORCID: 0009-0008-8448-1820)

[b]College of Wildlife and Protected Area, Northeast Forestry University, Harbin 150040, China; CL (linc110792@gmail.com, ORCID: 0009-0002-8998-2874); ZW (25252170@qq.com, ORCID: 0009-0008-1327-359X)

[c]Guangxi Zhuang Autonomous Region Forest Inventory and Planning Institute, Nanning 530011, China; ZD (yamasun.dzw817@163.com, ORCID: 0009-0000-8323-8441)

CONTACT Zhangwen Deng: yamasun.dzw817@163.com; Guangxi Zhuang Autonomous Region Forest Inventory and Planning Institute, Nanning 530011, China

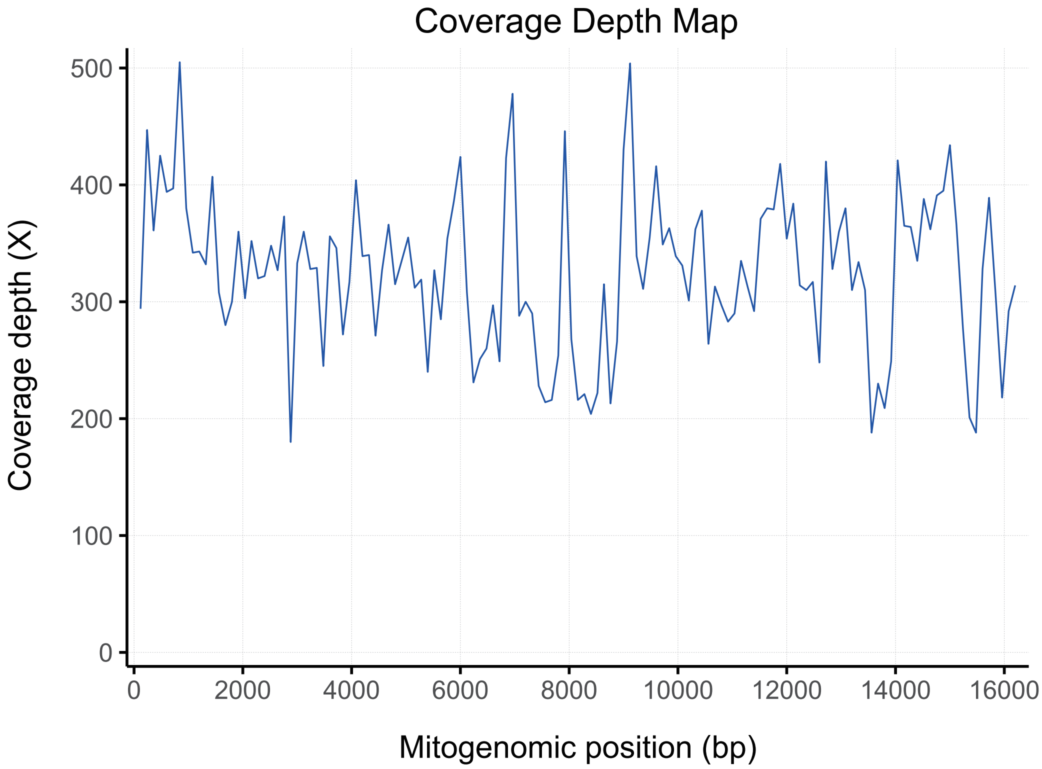

(1) Total genome length = 16,262 bp
(2) Average depth = 325.44 x
(3) Maximal depth = 505 x
(4) Minimal depth = 180 x

Supplementary Figure S1. Coverage depth plot for *Proedromys bedfordi* mitochondrial genome.
X and Y axis present nucleotide position of *P. bedfordi* mitochondrial genome and coverage depth, respectively.
